# Enhancement Methods of Hydropower Unit Monitoring Data Quality Based on the Hierarchical Density-Based Spatial Clustering of Applications with a Noise–Wasserstein Slim Generative Adversarial Imputation Network with a Gradient Penalty

**DOI:** 10.3390/s24010118

**Published:** 2023-12-25

**Authors:** Fangqing Zhang, Jiang Guo, Fang Yuan, Yuanfeng Qiu, Pei Wang, Fangjuan Cheng, Yifeng Gu

**Affiliations:** 1 Intelligent Power Equipment Technology Research Center, Wuhan University, Wuhan 430072, China; zfq@whu.edu.cn (F.Z.); w_sara@whu.edu.cn (P.W.); chengfj@whu.edu.cn (F.C.); guyifeng@whu.edu.cn (Y.G.); 2 School of Power and Mechanical Engineering, Wuhan University, Wuhan 430072, China; 3 School of Water Resources and Hydropower Engineering, Wuhan University, Wuhan 430072, China; yfqiu@whu.edu.cn

**Keywords:** HDBSCAN, WSGAIN, GP, hydropower unit, condition monitoring data, data enhancement

## Abstract

In order to solve low-quality problems such as data anomalies and missing data in the condition monitoring data of hydropower units, this paper proposes a monitoring data quality enhancement method based on HDBSCAN-WSGAIN-GP, which improves the quality and usability of the condition monitoring data of hydropower units by combining the advantages of density clustering and a generative adversarial network. First, the monitoring data are grouped according to the density level by the HDBSCAN clustering method in combination with the working conditions, and the anomalies in this dataset are detected, recognized adaptively and cleaned. Further combining the superiority of the WSGAIN-GP model in data filling, the missing values in the cleaned data are automatically generated by the unsupervised learning of the features and the distribution of real monitoring data. The validation analysis is carried out by the online monitoring dataset of the actual operating units, and the comparison experiments show that the clustering contour coefficient (SCI) of the HDBSCAN-based anomaly detection model reaches 0.4935, which is higher than that of the other comparative models, indicating that the proposed model has superiority in distinguishing between the valid samples and anomalous samples. The probability density distribution of the data filling model based on WSGAIN-GP is similar to that of the measured data, and the KL dispersion, JS dispersion and Hellinger’s distance of the distribution between the filled data and the original data are close to 0. Compared with the filling methods such as SGAIN, GAIN, KNN, etc., the effect of data filling with different missing rates is verified, and the RMSE error of data filling with WSGAIN-GP is lower than that of other comparative models. The WSGAIN-GP method has the lowest RMSE error under different missing rates, which proves that the proposed filling model has good accuracy and generalization, and the research results in this paper provide a high-quality data basis for the subsequent trend prediction and state warning.

## 1. Introduction

As the significance of hydropower units in modern power systems grows, the urgency for accurate condition monitoring and prediction increases. However, the quality of monitoring data in hydropower units is often compromised due to interference, abnormalities or failures in data acquisition and transmission links, leading to issues like data anomalies and missing data. These problems hamper the accuracy and reliability of condition monitoring data [1].

Traditional clustering methods such as K-Means [2,3,4,5,6,7,8], subspace clustering [9,10,11,12] and Mean-Shift [13,14,15,16] struggle to adapt to the varying distributions of monitoring and collection data in industrial settings. Furthermore, statistical feature-based methods like the Lyddane criterion [17,18] and the quartile method [19,20,21] are limited in their accuracy due to the fluctuating operating conditions of hydropower units, which affect the signal amplitude. Density-based clustering methods, such as Density-Based Spatial Clustering with Noise Applications (DBSCAN) [22,23,24], the Local Outlier Factor Algorithm (LOF) [25,26], Density Estimation Based Clustering (DENCLUE) [27], hierarchical agglomerative clustering (HAC) [28] and On-Point Sorting Clustering Methods (OPTICS) [29,30], although capable of extracting clusters of arbitrary shapes and recognizing noise, still face challenges in parameter selection and do not fully meet the current needs. The Hierarchical Density-Based Spatial Clustering with Noise Applications (HDBSCAN) [31,32,33,34,35,36], which integrates the advantages of these methods and addresses the parameter selection issue, has shown promising results in application verification.

The condition monitoring of missing values has a great impact on both dimensionality reduction analysis and model training, and the timely handling of missing data is crucial for intelligent decision making [1,37]. Data augmentation is a technical tool for generating new data samples by transforming or processing the original data while keeping the data labels unchanged. It is widely used in the fields of machine learning and deep learning to enhance the generalization ability of models and mitigate the overfitting problem, especially in the case of data scarcity, which is of great practical significance. Missing data filling is an important task in data processing, the goal of which is to estimate or fill the missing values in a dataset by a reasonable method to improve the availability and quality of the dataset. In recent years, traditional statistical methods, machine learning-based methods, time series data filling methods, multiple filling methods and so on have been mainly developed.

Traditional data filling methods, including mean filling and local interpolation, often fail to account for temporal dependencies between samples, resulting in a suboptimal filling accuracy. Adhikari et al.’s comprehensive survey [37] underscores the importance of accurate data handling in IoT for intelligent decision-making, specifically highlighting the inadequacy of many methods in addressing temporal correlations. Little and Rubin [38] illuminate the utility of mean filling in missing data analysis, yet also point out its limitations in maintaining the inherent structure and relationships within datasets. Ratolojanahary et al. [39] demonstrate improvements in multiple imputation for datasets with high rates of missingness but note the challenge of capturing temporal dependencies. Goodfellow et al. [40], in their pioneering work on GANs, opened avenues for data augmentation but faced challenges with unstable training, impacting the quality of imputed data. Kim et al. [41] discuss the potential of deep learning methods, including GANs, in complex data structures, yet identify overfitting and the neglect of time-related characteristics as issues. Haliduola et al. [42] approach missing data imputation in clinical trials using clustering and oversampling to enhance neural network performance but do not fully address the temporal aspects of data series. Zhang et al. [43] show progress in handling sequential data for time series imputation yet struggle with accurately reflecting temporal dynamics in variable datasets. Verma and Kumar [44] acknowledge the importance of capturing temporal sequences in healthcare data using LSTM networks but also highlight the computational complexities involved. Azur et al. [45] explore multiple imputation methods, revealing their limitations in handling datasets with significant time series elements. Finally, Song and Wan [46] highlight the effectiveness of certain interpolation methods in specific contexts but indicate their general inadequacy in capturing time-dependent data patterns. Together, these studies contribute to our understanding of the challenges in data imputation, particularly in capturing temporal dependencies, which is crucial for advancing the field. These studies collectively illustrate that while traditional data filling methods have evolved and improved in various ways, a significant gap remains in accurately addressing the temporal dependencies in data series. This gap underscores the need for more advanced and nuanced approaches to data imputation.

To overcome these limitations, Generative Adversarial Networks (GANs) [47,48,49,50] have been introduced in the field of data generation, capable of learning data distributions and generating synthetic data with features of real data. GANs can learn the distribution of data and generate synthetic data with real data features. In the data augmentation field, many studies have shown that GANs can be used to generate more data samples. Specifically, GANs can be trained on the original dataset to obtain a generative model. Then, the generative model is used to generate more data samples, which can be used to augment the original dataset, increase the number of data samples and improve the model’s generalization ability. However, traditional GANs have problems such as unstable training and mode collapse during the training process. To overcome the issues of traditional GANs, Wasserstein Generative Adversarial Networks (WGANs) were introduced [51,52,53]. By introducing the Wasserstein distance to measure the distance between generated data and real data, WGANs improve training stability and the quality of generated data. Furthermore, to increase the diversity of generated data, Gradient Penalty was introduced into WGAN, forming Wasserstein Generative Adversarial Networks with Gradient Penalty (WGAN-GP) [54,55,56,57]. Based on the GAN and WGAN architectures, dedicated generative imputation networks were developed for data imputation, namely, the Generative Adversarial Imputation Network (GAIN) [58] and Wasserstein Generative Adversarial Imputation Network (WGAIN) [59]. The Slim Generative Adversarial Imputation Network (SGAIN), a lightweight generative imputation network architecture without a hint matrix, was proposed as an improvement on the GAIN. To address the issues of traditional GANs in SGAIN, the Wasserstein Slim Generative Adversarial Imputation Network (WSGAIN) was further improved, along with the Wasserstein Slim Generative Adversarial Imputation Network with Gradient Penalty (WSGAIN-GP) [59].

In this study, we introduce a novel approach for enhancing the quality and usability of the condition monitoring data of hydroelectric units, addressing the limitations of traditional methods, such as inadequate accuracy, disregard for temporal dependencies and obscured data distribution characteristics. Our methodology uniquely integrates two advanced data processing techniques: anomaly detection using the HDBSCAN clustering method and data imputation through the WSGAIN-GP generative model. This combination not only retains the intrinsic characteristics of the data but also significantly improves their completeness and utility. The HDBSCAN clustering method effectively groups monitoring data according to density levels, enabling the precise identification of outliers, which is crucial for accurate data enhancement. Following this, the WSGAIN-GP generative model, utilizing unsupervised self-learning, adeptly approximates the distribution characteristics of real monitoring data. This is instrumental in generating high-quality substitutes for missing data, thereby addressing the gap left by traditional methods. Our contribution is noteworthy in that we are the first to apply these sophisticated methods to the realm of hydropower unit condition monitoring. By doing so, we not only preserve the fidelity of the data but also augment its integrity and applicability. The enhanced data quality and accuracy provided by our approach lay a solid foundation for the more reliable condition monitoring and prediction of hydropower units. This advancement is a step forward in realizing intelligent warnings for hydropower unit conditions, ultimately contributing positively to the maintenance and operational efficiency of these units. This paper delves into the specifics of our quality enhancement methodology, using the HDBSCAN clustering method and the WSGAIN-GP generative model, and presents experimental evidence demonstrating its significant impact on improving data quality and accuracy in hydroelectric unit condition monitoring.

## 2. Related Theory and Methods

### 2.1. HDBSCAN Clustering Approach

**Figure 1:**
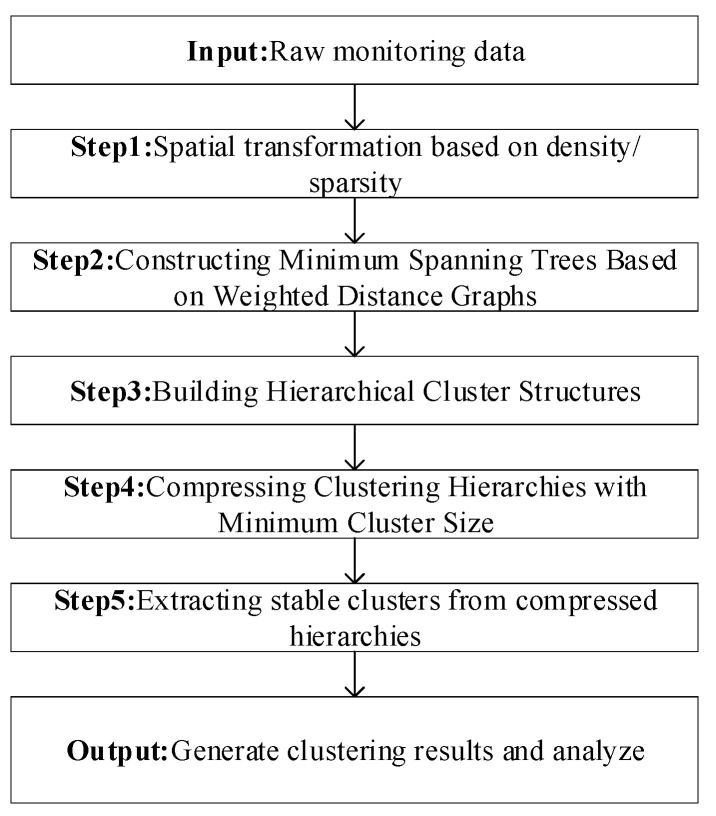
HDBSCAN algorithm flow chart.

HDBSCAN (Hierarchical Density-Based Spatial Clustering of Applications with Noise), an extension of the DBSCAN algorithm proposed by Campello et al. [60,61], is a density-based clustering method particularly effective for datasets with varying densities. Unlike DBSCAN, which relies on a uniform density threshold across the dataset, HDBSCAN identifies clusters of different densities by constructing a density tree, providing robustness against noise and outliers. The workflow of the HDBSCAN algorithm is illustrated in Figure 1.

The HDBSCAN algorithm follows these steps [32,33,34,35]:

Definition: The core distance $core_{k}\left(x\right)$ represents the distance from the current point x to its *k*-th nearest neighbor. For a border point (BP), $core_{k}\left(x\right)$ is infinite because $\left|N_{Eps}^{D}\left(x\right)\right|<MinPts$ in x’s Eps, making it meaningless. For a core point (CP), $core_{k}\left(x\right)$ is the minimum radius that ensures exactly MinPts samples in x’s Eps, i.e., the Euclidean distance between point x and the k-th nearest neighbor ($N^{k}\left(x\right)$) that satisfies $\left|N_{Eps}^{D}\left(x\right)\right|\geq MinPts$. min_cluster_size defines the minimum number of samples for a cluster. If the number of samples in a formed cluster is below this threshold, it is considered a noise (outlier) point. min_sample represents the minimum number of samples that must be included in the neighborhood of one point when calculating the density and minimum distance. min_cluster_size and min_sample are hyperparameters that need to be set for the HDBSCAN algorithm, as shown in Equations (1) and (2).

(1) $$core_{k}\left(x\right)=d\left(x,N^{k}\left(x\right)\right)$$

(2) k=min_sample

Reachable Distance (RD): Point x is a CP, and p is any point. The RD between x and p, denoted as d_$d_{k}^{RD}\left(x,p\right)$, is the maximum value between the core distance $core_{k}\left(x\right)$ of x and the Euclidean distance between x and p, as shown in Equation (3).

(3) $$d_{k}^{RD}\left(x,p\right)=\max\left(core_{k}\left(x\right),d\left(x,p\right)\right)$$

Mutual Reachable Distance (MRD): MRD requires both points x and p to be CPs; otherwise, it is meaningless. It represents the maximum value between the core distances $core_{k}\left(x\right)$ and $core_{k}\left(p\right)$ of the two points and the Euclidean distance between them, as shown in Equation (4).

(4) $$d_{k}^{MRD}\left(x,p\right)=\max\left\{core_{k}\left(x\right),core_{k}\left(p\right),d\left(x,p\right)\right\}$$

By transforming the dataset in space, the distances between dense points are reduced, while the distances involving sparse points are enlarged.

Step 1. Space Transformation: Utilizing density estimation to segregate low-density data from high-density data, reducing the impact of noise.

Step 2. Minimum Spanning Tree Construction: Building a tree graph from the weighted graph of transformed data points.

Step 3. Build a Hierarchical Clustering Structure: Creating a hierarchical structure by sorting and categorizing the edges in the tree.

Step 4. Tree Pruning and Compression: Limiting clusters based on min_cluster_size, refining the tree structure.

Step 5. Cluster Extraction: Selecting the most stable clusters based on local density estimates and cluster stability calculations. The goal of density-based clustering algorithms is to find the region with the highest density. The local density effective estimate λ for point x can be represented by the reciprocal of $core_{k}\left(x\right)$, as shown in Equation (5).

(5) $$\lambda =\frac{1}{core_{k}\left(x\right)}$$

$\lambda_{max}^{C_{i}}\left(x\right)$ denotes the maximum λ value of point x departing from cluster $C_{i}$, and $\lambda_{min}^{C_{i}}\left(x\right)$ represents the minimum λ value of point x belonging to cluster $C_{i}$. The stability $\sigma (C_{i})$ of cluster $C_{i}$ is defined as shown in Equation (6):

(6) $$\sigma (C_{i})=\sum_{x\in C_{i}}\left(\lambda_{max}^{C_{i}}\left(x\right)-\lambda_{min}^{C_{i}}\left(x\right)\right)$$

Select the final data clusters based on stability, generate clustering results and identify outlier points based on the clustering results.

The calculation formula for the data missing rate is defined as Equation (7):

(7) $$\delta =1-\frac{n}{N}$$

where n is the number of existing data samples, and N is the number of data samples that should exist based on the date–time interval and sampling storage interval.

Since it is difficult to directly determine whether the measured data are an abnormal sample, in order to objectively evaluate the effect of anomaly detection, the Silhouette Coefficient Index (SCI) is used as a quantitative evaluation index. The SCI allows for a quantitative comparison of clustering results from the perspective of data distribution in cases lacking support from true data labels. It is defined as Equation (8):

(8) $$SCI=\frac{1}{N}\sum_{i=1}^{N}\frac{y_{i}-x_{i}}{max\left(x_{i},y_{i}\right)}$$

where $x_{i}$ represents the average distance between the i-th sample and other samples in the same cluster, reflecting the cohesion of samples within the cluster. $y_{i}$ represents the average distance between the i-th sample and all samples in the nearest neighboring cluster, reflecting the separation between clusters. SCI∈[−1, 1] and a larger SCI value indicate a smaller intra-cluster distance and larger inter-cluster distance, representing a better clustering effectiveness.

### 2.2. WSGAIN-GP Algorithm

The development of WSGAIN-GP as an advanced tool for data estimation and imputation is grounded in the progressive evolution of Generative Adversarial Networks (GAN) and their variants. This method is an extension of the foundational GAIN model, further refined by subsequent iterations such as SGAIN and WSGAIN, culminating in a sophisticated approach for data imputation.

Originally, the GAIN network introduced a generator to create missing data and a discriminator to distinguish between real and imputed data. This adversarial training process involves the discriminator minimizing classification loss, while the generator aims to maximize the misclassification rate of the discriminator. In this framework, GAIN’s discriminator receives additional data through a ‘hint’ mechanism, albeit at the cost of increased computational demands. SGAIN, a more streamlined version of GAIN, eliminates the Hint Generator and the associated Hint Matrix, thereby simplifying the architecture [62,63]. This approach adopts a two-layer neural network structure for both the generator and discriminator, in contrast to GAIN’s three-layer setup, as detailed by Goodfellow et al. [40].

**Figure 2:**
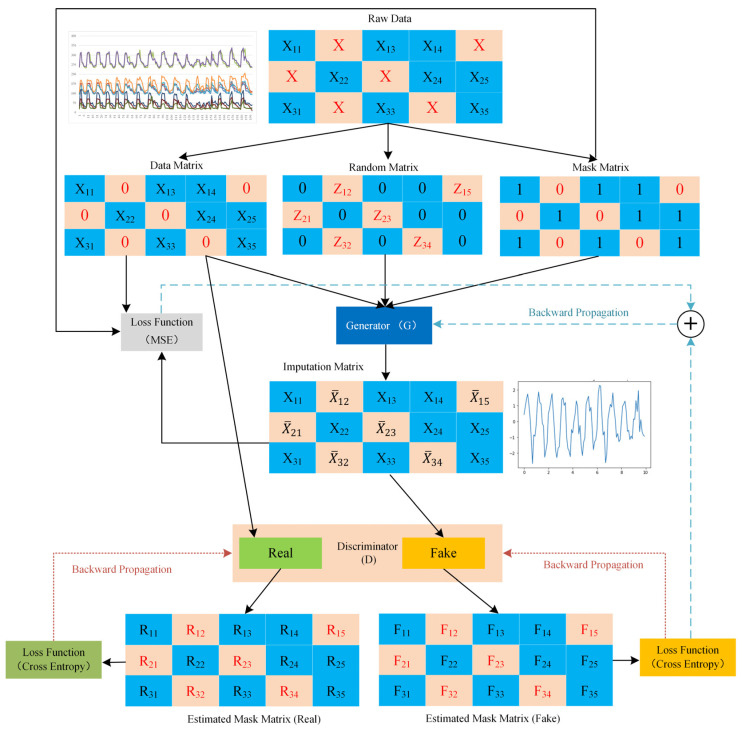
Structure of the WSGAIN-GP network.

Building upon SGAIN, WSGAIN addresses challenges such as pattern collapse and gradient vanishing. It does so by incorporating the Wasserstein distance to measure discrepancies between real and generated data, thereby enhancing the stability of the training process. Further improving upon WSGAIN, the WSGAIN-GP model introduces a gradient penalty technique, moving away from weight clipping. This modification, as part of the loss function, enhances the overall efficacy of the network [59]. The network architecture of WSGAIN-GP is depicted in Figure 2 of the manuscript.

Assuming a d-dimensional spatial dataset $\chi =\left\{\chi_{1},\chi_{2},\cdots ,\chi_{d}\right\}$, the data vector $X=\left(X_{1},X_{2},\cdots ,X_{d}\right)$ is a random variable taking values in the dataset χ, and its distribution is defined as P(X). The mask vector $M=\left(M_{1},M_{2},\cdots ,M_{d}\right)$ is a random vector taking values in ${\left\{0,1\right\}}^{d}$. Define a new data space $\tilde{\chi }=\left\{{\tilde{\chi }}_{1},{\tilde{\chi }}_{2},\cdots ,{\tilde{\chi }}_{d}\right\}$ and a new random variable $\tilde{X}=\left({\tilde{X}}_{1},{\tilde{X}}_{2},\cdots ,{\tilde{X}}_{d}\right)\in \tilde{\chi }$. Then, as shown in Equation (9):

(9) $${\tilde{X}}_{i}=\{\begin{array}{c} X_{i},\;\;M_{i}=1\\ {}*,\;M_{i}=0 \end{array}$$

where * does not belong to any $X_{i}$ and represents an unobserved value. Therefore, the mask vector M indicates which elements of X have been observed, and M can be used to recover X. Define the dataset $D=\left\{\left({\tilde{x}}^{i},m^{i}\right)\right\}$, where ${\tilde{x}}^{i}$ is a copy of $\tilde{X}$ and $m^{i}$ corresponds to the recovered M. The goal of data estimation imputation is to supplement every unobserved value in ${\tilde{X}}_{i}$ based on the conditional distribution $P(X|\tilde{X}={\tilde{x}}_{i})$. Define $\bar{X}$ as the output vector estimated for each ${\left\{X_{i}\right\}}_{1}^{d}$ and $\hat{X}$ as the final estimated result vector, as shown in Equation (10).

(10) $$\hat{X}=M\cdot X+\left(1-M\right)\cdot \bar{X}$$

Generator (G) Model: The generator (G) takes M and random noise variable Z as inputs and outputs the estimated matrix $\bar{X}$. Z is a d-dimensional variable, $Z=\left(Z_{1},\cdots ,Z_{d}\right)$, where each ${\left\{Z_{i}\right\}}_{1}^{d}$ has non-missing values from ${\left\{X_{i}\right\}}_{1}^{d}$, and missing values in ${\left\{X_{i}\right\}}_{1}^{d}$ are replaced by random noise values. $N=\left(N_{1},\cdots ,N_{d}\right)$ represents an output of a function that samples random values from a continuous uniform distribution, commonly configured to use the interval [−0.01,+0.01], as shown in Equations (11) and (12):

(11) Z=M⋅X+(1−M)⋅N

(12) $$\bar{X}=G\left(M,Z\right)$$

The loss function L(G) is expressed as shown in Equation (13[M1] ):

(13) $$L\left(G\right)=\nabla_{\theta_{G}}-\frac{1}{mb}\sum_{j=1}^{mb}\left[\left(1-\tilde{m}\left(j\right)\right)\cdot D\left(\bar{x}\left(j\right)\right)\right]+\frac{\alpha }{mb}\sum_{j=1}^{mb}\left[\tilde{m}\left(j\right)\cdot {\left(\tilde{x}\left(j\right)-\bar{x}\left(j\right)\right)}^{2}\right]$$

Discriminator (D) Model: The discriminator (D) is a crucial component in adversarial games. Its task is to receive samples from the generator or from the real dataset and attempt to classify them as either real samples or fake samples (samples generated by the generator). The goal is to correctly classify samples, i.e., accurately distinguish between real and generated samples. Training improves the discriminator’s ability to discern real from fake. Since the goal of WSGAIN-GP is to eliminate weight clipping due to weight trimming [64], there is no weight trimming. To improve training, a gradient penalty is included as a component of the loss function L(D), as shown in Equations (14) and (15).

(14) $$\dot{x}\left(j\right)=\tilde{m}\left(j\right)\cdot \left(\tilde{\epsilon }\left(j\right)\cdot \tilde{x}\left(j\right)\right)+\left(\left(1-\tilde{m}\left(j\right)\right)\cdot \left(1-\tilde{\epsilon }\left(j\right)\right)\cdot \bar{x}\left(j\right)\right)$$

(15) $$L\left(D\right)=\nabla_{\theta_{D}}\frac{1}{\mathrm{mb}}\sum_{j=1}^{\mathrm{mb}}\left[\tilde{m}\left(j\right)\cdot D\left(\tilde{x}\left(j\right)\right)]-\frac{1}{\mathrm{mb}}\sum_{j=1}^{\mathrm{mb}}[\left(1-\tilde{m}\left(j\right)\right)\cdot D\left(\bar{x}\left(j\right)\right)\right]+\frac{\lambda }{\mathrm{mb}}\sum_{j=1}^{\mathrm{mb}}{\left(\|\nabla_{\dot{x}\left(j\right)}C{\left(\dot{x}\left(j\right)\right)\|}_{2}-1\right)}^{2}$$

The detailed steps of the WSGAIN-GP algorithm are shown in Algorithm 1 [59].
**Algorithm 1**. Pseudo-code of the WSGAIN-GP algorithm1**Input**: X                    //Data sets with missing values 2**Output**: $\hat{X}$                    //The populated dataset3**Parameters:** mb*,* α*,*clip*,*n_iter*,* n_critic4M ←mask(X) //Equation (9)5For iter ←1 to n_iter **do**6     For *extra* ←1 to n_critic **do**7          Draw mb samples from X ${\left\{{\tilde{x}}_{j}\right\}}_{j=1}^{d}$, M ${\left\{{\tilde{m}}_{j}\right\}}_{j=1}^{d}$, N ${\left\{{\tilde{n}}_{j}\right\}}_{j=1}^{d}$, ${\left\{{\tilde{\epsilon }}_{j}\right\}}_{j=1}^{d}$
8          For j ←1 to mb do9${\tilde{z}}_{j}\leftarrow {\tilde{m}}_{j}\cdot {\tilde{x}}_{j}+\left(1-{\tilde{m}}_{j}\right)\cdot {\tilde{n}}_{j}$10               
${\bar{x}}_{j}=G\left({\tilde{m}}_{j},{\tilde{z}}_{j}\right)$
11               $\dot{x}\left(j\right)=\tilde{m}\left(j\right)\cdot \left(\tilde{\epsilon }\left(j\right)\cdot \tilde{x}\left(j\right)\right)+\left(\left(1-\tilde{m}\left(j\right)\right)\cdot \left(1-\tilde{\epsilon }\left(j\right)\right)\cdot \bar{x}\left(j\right)\right)$ //Equation (14)12          End13          // Discriminator optimization: update the discriminator D by Adam or RMSprop or SGD14           
$\nabla_{\theta_{D}}\frac{1}{mb}\sum_{j=1}^{mb}{ℒ}_{D}\left(D\left(\tilde{x}\left(j\right)\right),D\left(\bar{x}\left(j\right)\right),\tilde{m}\left(j\right)\right)+\frac{1}{mb}\sum_{j=1}^{mb}{ℒ}_{GradPen}\left(D\dot{x}\left(j\right)\right)$
15     End16     //Generator optimization: updating the generator G via Adam or RMSprop or SGD17$\nabla_{\theta_{G}}-\frac{1}{mb}\sum_{j=1}^{mb}{ℒ}_{G}\left(D\left(\bar{x}\left(j\right),\tilde{m}\left(j\right)\right)\right)+\frac{\alpha }{mb}\sum_{j=1}^{mb}{ℒ}_{MSE}\left(\tilde{x}\left(j\right),\bar{x}\left(j\right),\tilde{m}\left(j\right)\right)$18End19Z=M⋅X+(1−M)⋅N20$\bar{X}=G\left(M,Z\right)$21$\hat{X}=M\cdot X+\left(1-M\right)\cdot \bar{X}$

## 3. Enhancement Methodology Flow of Hydropower Unit Condition Monitoring Data Based on HDBSCAN-WSGAIN-GP

**Figure 3:**
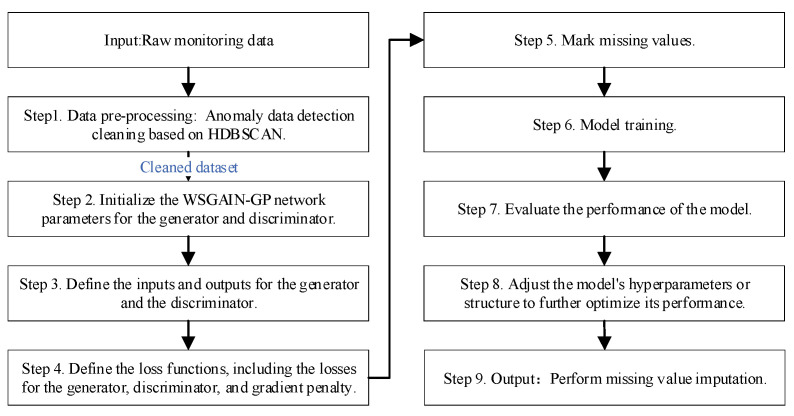
Quality Enhancement Process of Hydropower Unit Monitoring Data Based on HDBSCAN-WSGAIN-GP.

A monitoring data quality enhancement method based on HDBSCAN-WSGAIN-GP improves the quality and usability of hydropower unit condition monitoring data by combining the advantages of density clustering and a generative adversarial network. The quality enhancement process of hydropower unit monitoring data based on HDBSCAN-WSGAIN-GP is shown in Figure 3. The detailed steps are as follows:

Step 1. Data pre-processing: Anomaly data detection cleaning based on HDBSCAN.

Step 2. Initialize the network parameters for the generator and discriminator.

Step 3. Define the inputs and outputs for the generator and the discriminator.

Step 4. Define the loss functions, including the losses for the generator, discriminator and gradient penalty.

Step 5. Mark missing values and construct mask vector M.

Step 6. During model training, alternate between training the generator network and the discriminator network, updating model parameters by optimizing the loss functions, which include both the generator and discriminator losses. The generator is used to generate estimated values for imputing missing data, while the discriminator evaluates the difference between generated data and real data.

Step 7. After each training epoch, evaluate the performance of the model, including the imputation effectiveness of the generator and the discrimination accuracy of the discriminator.

Step 8. Based on the evaluation results, adjust the model’s hyperparameters or structure to further optimize its performance, ultimately obtaining an efficient WSGAIN-GP model for imputing missing data.

Step 9. Perform missing value imputation: using the trained WSGAIN-GP model, merge the imputed data generated by the generator with the existing data in the original dataset to obtain a complete dataset, thereby achieving the imputation of missing data.

To quantitatively assess the consistency of the filled data sequence with the original data sequence in terms of the distribution and characteristics, KL Divergence, JS Divergence and Hellinger Distance are introduced to quantify the similarity between two distributions, as shown in Equations (16)–(18):

(16) $$KL\left(p∥q\right)=\sum p\left(i\right)\log\frac{p\left(i\right)}{q\left(i\right)}$$

(17) $$JS\left(p∥q\right)=\frac{1}{2}KL\left(p∥\frac{p+q}{2}\right)+\frac{1}{2}KL\left(q∥\frac{p+q}{2}\right)$$

(18) $$HD\left(p∥q\right)=\sqrt{\frac{1}{2}\sum {\left(\sqrt{p\left(i\right)}-\sqrt{q\left(i\right)}\right)}^{2}}$$

KL Divergence, JS Divergence and Hellinger Distance are all non-negative. KL Divergence ranges from 0 to ∞, while JS Divergence and Hellinger Distance range from 0 to 1. Smaller values of these three metrics indicate a greater similarity between two distributions, with a value of 0 indicating complete similarity.

## 4. Case Analysis

### 4.1. Data Collection

In order to validate the effectiveness of the proposed method, this study conducted an analysis using the actual operational monitoring data of a mixed-flow hydropower unit in a fengtan hydropower station in the Central China region. The data anomaly detection and data imputation methods were tested separately. The unit parameters are presented in Table 1.

**Figure 4:**
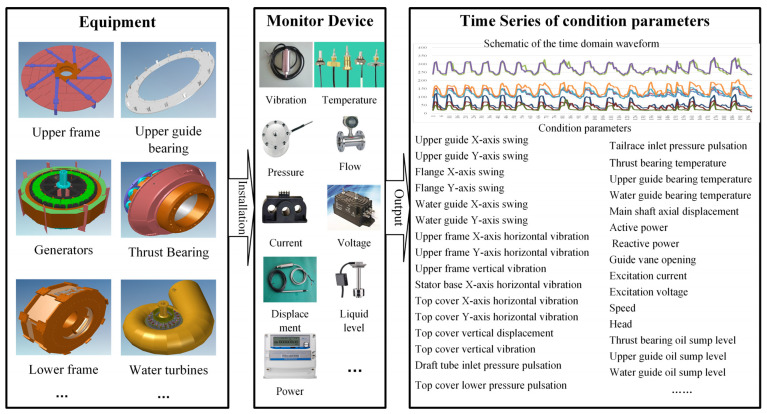
Time series of online monitoring data for hydropower units.

The unit is equipped with an online monitoring system, which utilizes monitoring equipment to continuously collect, monitor and automatically record various state parameters such as the vibration, deflection, pressure pulsation, air gap, stator temperature, oil level, active power, water level and rotational speed during the operation of the unit. This enables real-time access to the current operating status of the unit. The time series grouping of online monitoring data for this unit is illustrated in Figure 4.

**Figure 5:**
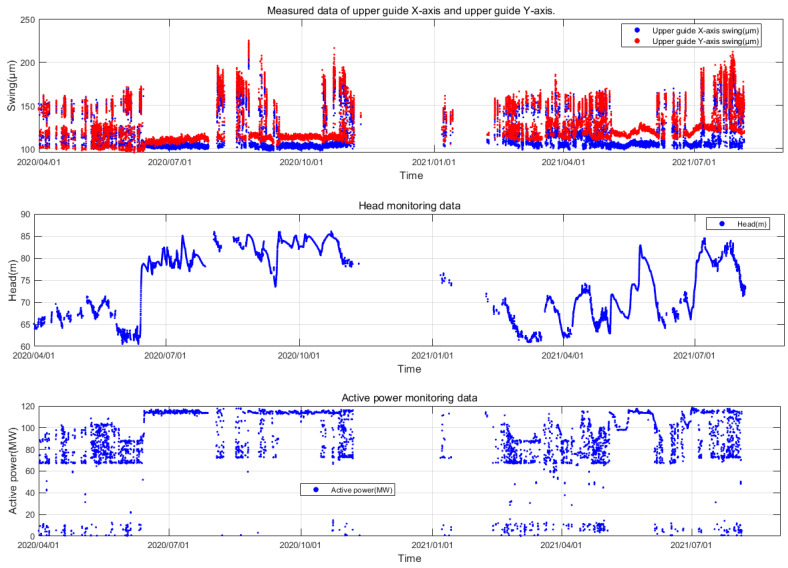
Unit Measurement Dataset from Fengtan Hydropower Station Unit 2.

To demonstrate the process of the proposed method and validate its effectiveness, this study has chosen the swing monitoring parameters as an example. The same procedure can be applied to other parameters. Specifically, we obtained the upper guide swing deflection data (S), corresponding head (H) and active power (P) of the unit from 3 April 2020 to 4 August 2021 from the unit’s condition online monitoring system of the Fengtan hydropower station. As illustrated in Figure 5, due to the characteristic of having a high sampling frequency but low data storage frequency in the online monitoring system of hydroelectric units, the actual time interval of the stored measured data is 15 min, amounting to a total of 26,245 samples. The overall missing rate δ of the original dataset is calculated to be 0.438. The missing data within the dataset are represented as NAN.

**Figure 6:**
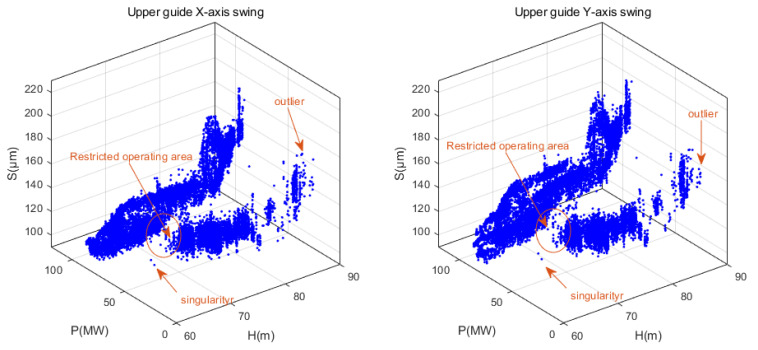
Three-dimensional dataset of upper guide swing and operating condition parameters from Fengtan Hydropower Station Unit 2.

From Figure 5, it can be observed that the amplitude variation pattern of the upper guide swing deflection (S) is complex. This complexity is attributed to its coupling with factors such as changes in operational parameters and noise. The water head (H) exhibits long-term low-frequency fluctuation characteristics, primarily influenced by seasonal changes in the external environment. The active power output of the unit (P) displays short-term high-frequency fluctuation characteristics. This is mainly due to the fact that the unit’s output is dynamically and flexibly adjusted in real time based on the load demand from the grid side. During this adjustment process, the vibration characteristics of the unit are taken into consideration, aiming to avoid operating conditions with intense vibrations. Therefore, when conducting anomaly detection for unit parameters, it is imperative to consider the coupled analysis of the actual operational characteristics of the unit. In this study, the influence of operational parameters H and P on the operational state parameter S is simultaneously considered. By combining these operational parameters, the measured data of the hydroelectric unit are structured into a three-dimensional dataset $\Omega_{0}=\left(H,P,S\right)$, as illustrated in Figure 6.

### 4.2. Anomaly Detection

**Figure 7:**
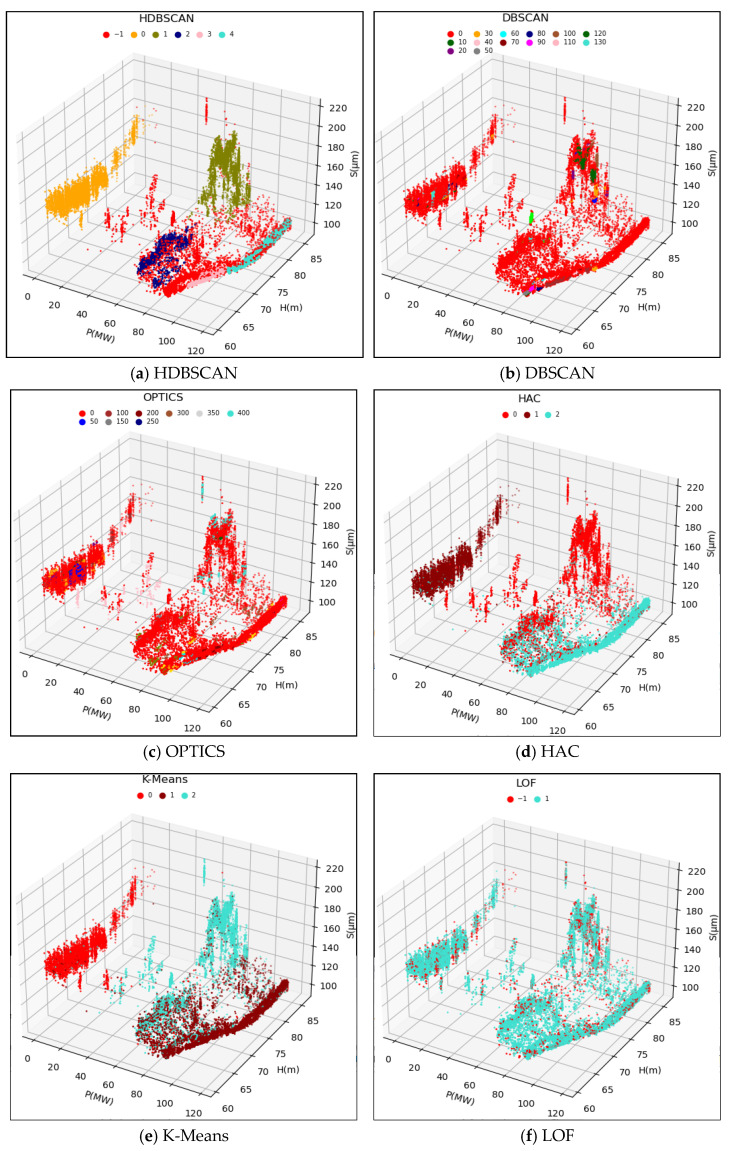
Clustering results of different anomaly detection methods for the upper guide X-axis swing.

**Figure 8:**
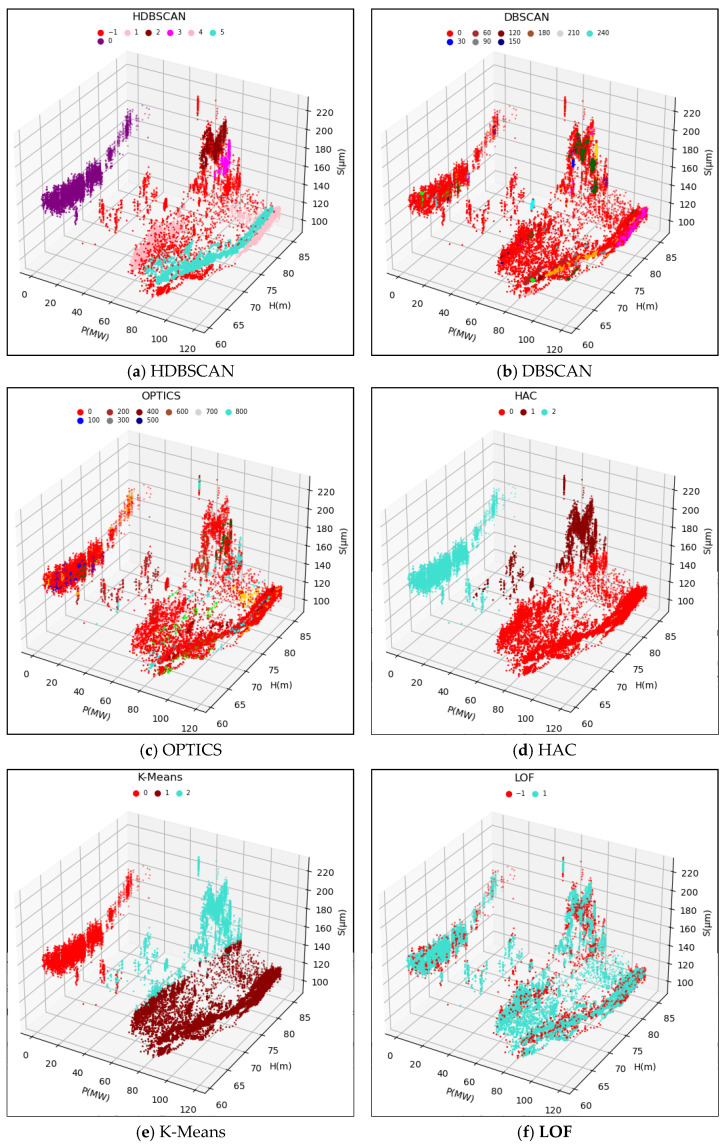
Clustering results of different anomaly detection methods for the upper guide Y-axis swing.

The original data samples were fed into the HDBSCAN model, and the computed results are depicted in Figure 7a and Figure 8a. The HDBSCAN method adaptively identified effective data clusters and marked outliers, including singular points and anomalous points, as noise points. By removing these noise points from the original data samples, a denoised and valid dataset $\Omega_{1}=\left(H_{1},P_{1},S_{1}\right)$ was obtained. This denoised dataset will be further input into the WSGAIN-GP model for missing data imputation.

To validate the effectiveness of the HDBSCAN anomaly detection method, this study compared the results with those of other methods such as DBSCAN, OPTICS, LOF, HAC and K-Means. Based on the methodology principles and references [24,25,26,27,28,29,30], some of the initial parameters for these comparative methods are shown in Table 2. The performance of different methods is evaluated in terms of the silhouette coefficient index, and the parameters are optimally tuned by a grid search method.

The clustering results of the different methods compared are shown in Figure 7 and Figure 8 as (b)~(f), respectively.

Different detection methods are used to detect anomalies for the 3D dataset $\Omega_{X}=\left(H,P,S_{X}\right)$, consisting of a head, active and upward-guided X-axis swing, and $\Omega_{Y}=\left(H,P,S_{Y}\right)$, consisting of a head, active and upward-guided Y- axis swing, respectively, and the computation of the Profile Coefficient Indicator (SCI) standardized example is shown in Table 3.

Combined with the clustering effect graph and the contour coefficient comparison data table, the HDBSCAN method has the highest contour coefficient in the comparison of the two dataset calculations, and at the same time, it can detect and recognize the effective data and abnormal data better, and the clusters divided into clusters are more in line with the actual operating conditions.

### 4.3. Data Filling

The missing rate $\delta_{1}=0.492$ of the noise reduced dataset $\Omega_{1}$ detected by the HDBSCAN method and the noise reduced dataset are further input into the trained WSGAIN-GP model for missing value filling.

The literature [56] shows that when the missing rate is greater than 50%, the accuracy of the WSGAIN-GP method of filling is significantly greater than that of other methods such as KNN and spline interpolation. According to the literature and several experiments, the network parameters of the WSGAIN-GP model involving the generator and the adversary are shown in Table 4.

The initialization assignments of the main parameters of the WSGAIN-GP model are shown in Table 5

**Figure 9:**
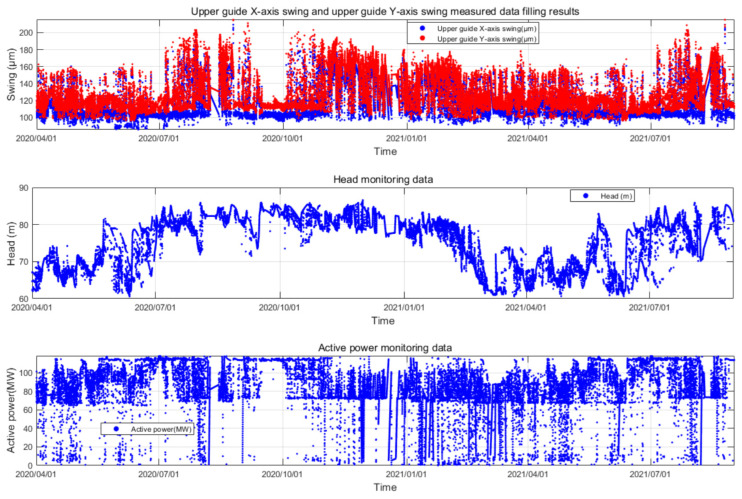
Results of WSGAIN-GP filled head, active and upward-guided swing.

Based on the model training, incomplete data sequences were input for data filling, and the results of the WSGAIN-GP-filled Upper Guide swing, head and active are shown in Figure 9.

**Figure 10:**
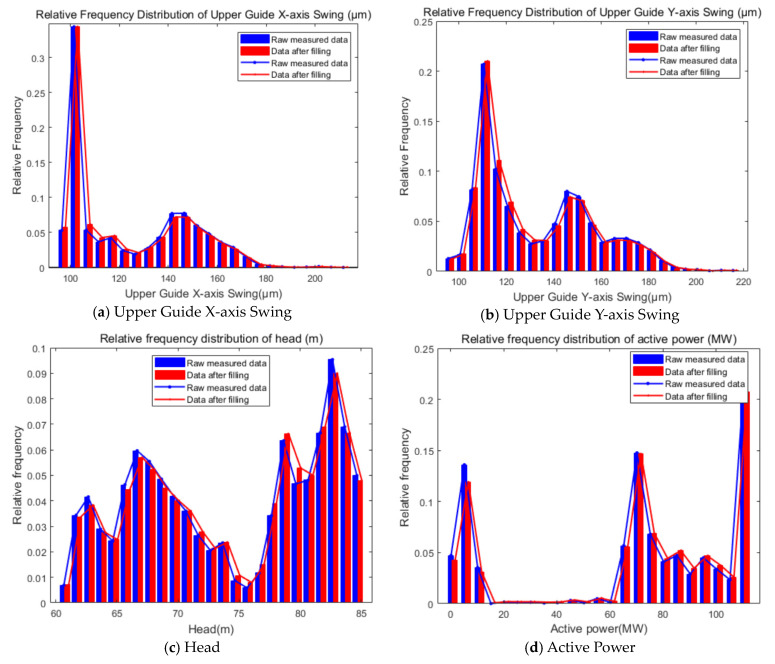
Relative frequency distributions after filling in the enhancement with measured data.

The relative frequency distribution of the measured data after filling the enhancement is shown in Figure 10.

Combined with the filling results in Figure 9 and Figure 10, it can be seen that the distribution of the data after filling by the WSGAIN-GP model is closer to the distribution of the measured values, which indicates that the filling method is able to better safeguard the data characteristics and distribution, and the quantitative indexes of the differences in the data distribution are shown in Table 6. The KL dispersion, JS dispersion and Hellinger distance of the data distributions before and after the filling of the upper guide swing, head and active power are close to 0, which indicates that the WSGAIN-GP filling model is able to learn the distribution and characteristics of the real data better and guarantees the accuracy of the generated data.

**Figure 11:**
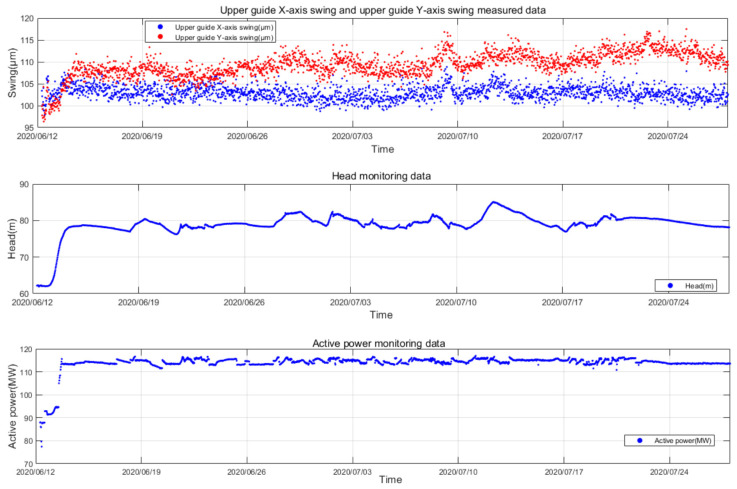
A complete short-term state sequence dataset for hydropower units.

In order to further validate the superiority of the proposed data filling method and at the same time study the performance of different data filling methods with different missing rates, the short-term complete state sequence dataset of hydropower units in the case study is selected for comparative analysis. For the comparison experimental dataset, the sampling interval is 30 min, and the sampling start and end time is from 7:00 p.m. on 12 June 2020 to 7:00 p.m. on 25 July 2020, with a total length of 43 days and a total of 2064 measured complete data samples, as shown in Figure 11. Non-complete sequences with different missing rates were generated by random missing, the missing rates were taken as 10%, 30%, 50% and 70%, respectively, considering the engineering reality, and the comparison methods were chosen as SGAIN, GAIN and KNN.

The Root Mean Square Error (RMSE) was used to measure the accuracy of the data filling results, as shown in Equation (19).

(19) $$RMSE=\sqrt{\frac{1}{N}\sum_{i=1}^{N}{\left[{\hat{y}}_{i}-y_{i}\right]}^{2}}$$

where *N* denotes the number of samples, ${\hat{y}}_{i}$ represents the filled value output by the model and $y_{i}$ denotes the measured true value.

**Figure 12:**
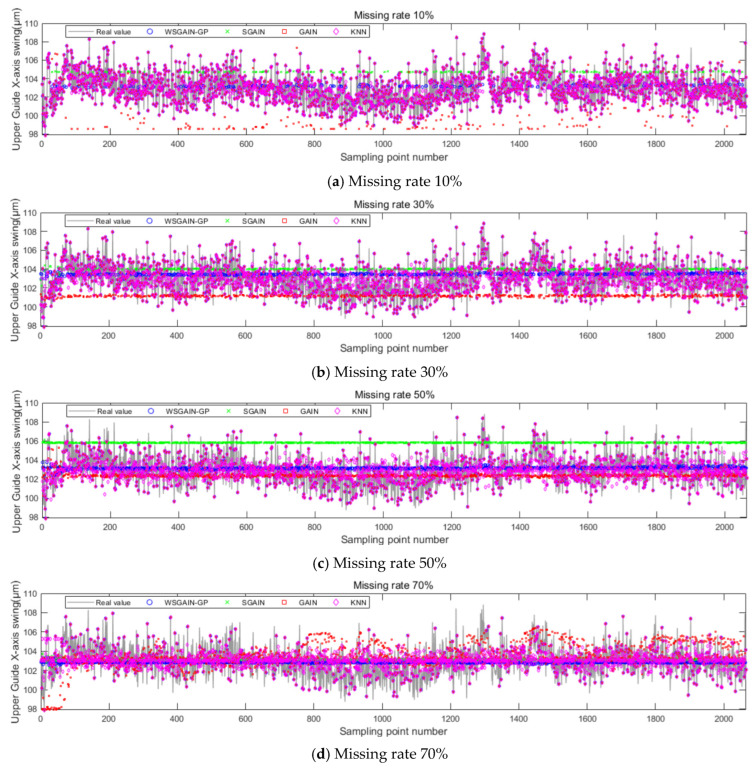
Filling results of each method for the upper guide X-axis swing at different missing rates.

**Figure 13:**
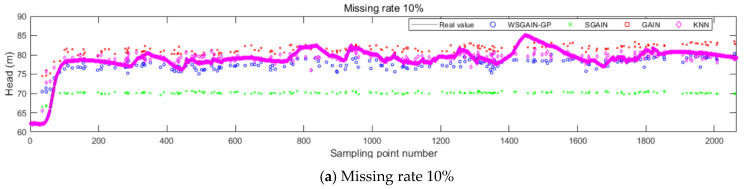

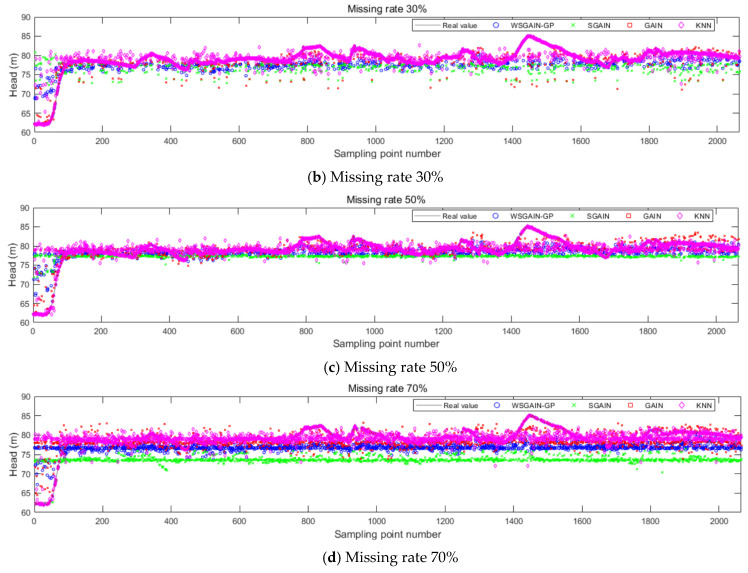
Filling results of each method for the head at different missing rates.

**Figure 14:**
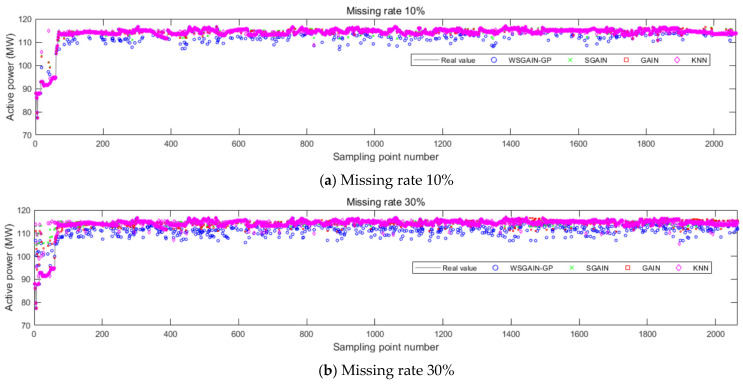

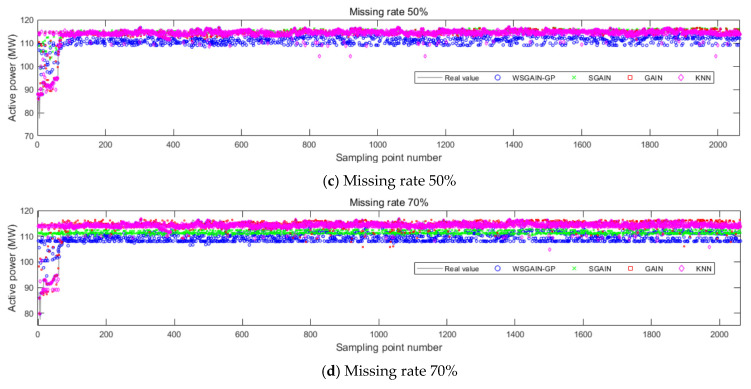
Filling results of each method for active power at different missing rates.

**Figure 15:**
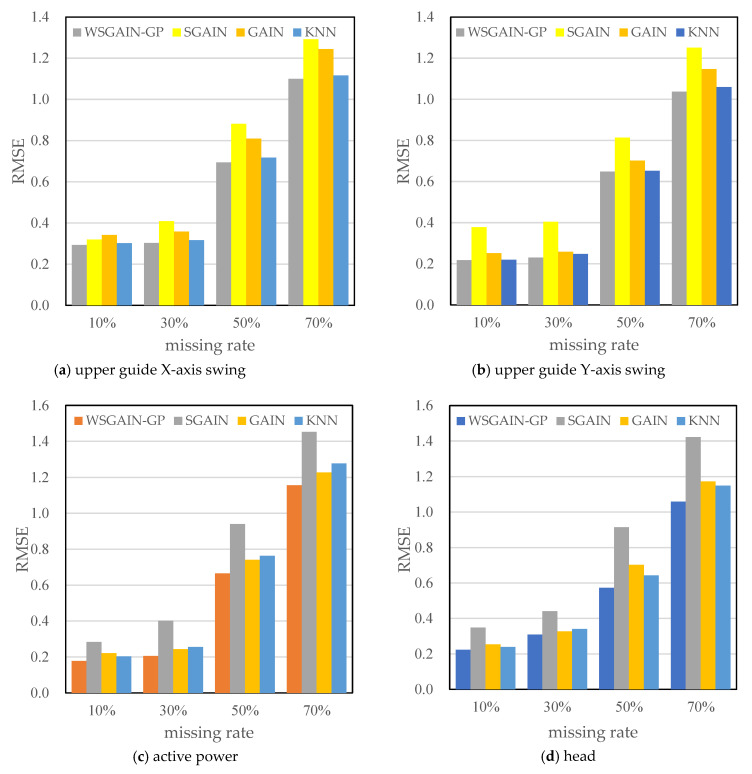
Comparison of filling errors (RMSE) for different methods.

The filling results of the different methods for different missing rates for the upper guide X-axis swing, head and active power of the hydroelectric units are shown in Figure 12, Figure 13, Figure 14 and Figure 15, and the horizontal X-axis serial numbers represent the serial numbers of the original samples in chronological order.

In order to eliminate the effect of non-complete sequence differences generated randomly according to the missing rate, set the number of repetitive trials to 1000 times, each time, respectively, according to a different missing rate-generated non-complete sequences, using different methods to fill, observe and record the error of each experiment and, ultimately, the average error of the 1000 experiments as the final error. The error results are shown in Table 7.

The statistical plots of the filling errors of each method for the upper guide X-axis swing, upper guide Y-axis swing, head and active power of the hydropower units are shown in Figure 14.

Based on the comparison with Figure 11, Figure 12, Figure 13 and Figure 14 and Table 7, it is evident that the WSGAIN-GP method for missing data imputation consistently yields the lowest error across various data missing rates, with an average reduction in the root mean square error of 0.0936, thereby demonstrating the highest accuracy. This method shows commendable effectiveness in filling gaps for hydroelectric unit state parameters with a high randomness, such as the pendulum swing, as well as operational parameters like the active power and head. The WSGAIN-GP model employs a generative adversarial network structure, wherein the interplay between the generator and discriminator approximates the distribution of real data. By generating data, the model is able to complete the missing parts of the monitoring data, resulting in a more complete and continuous dataset.

## 5. Conclusions

In addressing issues such as anomalies and missing data that compromise the quality of condition monitoring datasets in hydropower units, this chapter introduces a method for enhancing data quality through the integration of HDBSCAN-WSGAIN-GP. This method capitalizes on the strengths of density clustering and generative adversarial networks to enhance the reliability and utility of the condition monitoring data.

Initially, the HDBSCAN clustering method categorizes the monitoring data based on density levels, aligned with operational conditions, to adaptively detect and cleanse anomalies in the dataset. Furthermore, the WSGAIN-GP model, through its data imputation capabilities, employs unsupervised learning to understand and replicate the features and distribution patterns of actual monitoring data, thereby generating values for missing data.

The validation analysis, conducted using an online monitoring dataset from real operational units, provides compelling evidence of the method’s effectiveness:

(1) Comparative experiments reveal that the clustering contour coefficient (SCI) of the anomaly detection model based on HDBSCAN achieves 0.4935, surpassing those of other comparative models, thereby demonstrating its superior ability in distinguishing between valid and anomalous samples.

(2) The probability density distribution of the data imputation model based on WSGAIN-GP closely mirrors that of the measured data. Notably, the Kullback–Leibler (KL) divergence, Jensen–Shannon (JS) divergence and Hellinger’s distance metrics, when comparing the distribution between the imputed and original data, approach values near zero, indicating a high degree of accuracy in data representation.

(3) Through comparative analyses with other filling methods, including SGAIN, GAIN and KNN, the WSGAIN-GP model demonstrates superior effectiveness in data imputation across various rates of missing data. The Root Mean Square Error (RMSE) of the WSGAIN-GP consistently outperforms other models, particularly noted in its lowest RMSE across different missing data rates. This confirms the high accuracy and generalization capability of the proposed imputation model.

The findings and methodologies presented in this study lay a robust foundation for high-quality data, crucial for subsequent trend prediction and state warnings in the context of hydropower unit monitoring.

## Figures and Tables

**Table 1:** Parameter table of an experimental unit [65].

Unit Equipment	Parameter	Parameter Value
Hydraulic Turbine	Model	HLA898-LJ-419
	Rated output	117.5 MW
	Rated head	73 m
	Head range	57.5~86.2 m
	Runaway speed	300 r/min
	Rated flow	182 m³/s
	Rated speed	150 r/min
Generator	Model	SF115-40/8540
	Rated capacity	127.78 MVA
	Power factor	0.9
	Rated voltage	13.8 kV
	Rated speed	150 r/min
	Runaway speed	300 r/min
	Number of poles	40

**Table 2:** Initialization parameters for different detection methods.

Method	Initialization and Assignment of Main Parameters
DBSCAN	eps = 0.5, min_samples = 10
OPTICS	eps = 0.5, min_samples = 10
HAC	n_clusters = 3
K-Means	Use the Elbow Method to select the best K value, n_clusters = 3
LOF	n_neighbors = 20, contamination = 0.1
HDBSCAN	min_cluster_size = 920, min_samples = 7

**Table 3:** Comparison table of the results of different testing methods.

Method	$\mathrm{SCI}\;(\Omega_{X})$	$\mathrm{SCI}\;(\Omega_{Y})$
*DBSCAN*	0.3644	0.4169
OPTICS	0.2879	0.3998
HAC	0.3315	0.3521
K-Means	0.3490	0.3482
LOF	0.0098	0.0186
**HDBSCAN**	**0.4850**	**0.4935**

**Table 4:** WSGAIN-GP Network Structure Parameter Settings.

Type	Network Level	Network Name	Number of Neurons	Activation Function
Generator (G)	1	Full connection layer	32	Relu
	2	Full connection layer	32	tanh
Discriminator (D)	1	Full connection layer	32	Relu
	2	Full connection layer	32	-

**Table 5:** WSGAIN-GP Model Initialization Parameter Settings.

Parameter Name	Parameter Meaning	Parameter Value
clip_value	Gradient trimming value	0.01
n_critic	Additional training times for the discriminator	5
batch_size	Batch size	128
miss_rate	Missing rate	0.492
lambda_gp	Gradient penalty weight	10
n_iterations	Number of iterations	4000
α	Mean Square Error MSE Weighting Ratio	100
$l_{r}$	Learning rate	0.001
$\beta_{1}$	Exponentially weighted average of gradients, hyperparameters of Adam’s optimizer	0.9
$\beta_{2}$	Exponentially weighted average of squared gradients, hyperparameters of Adam’s optimizer	0.999
decay	Weight decay, hyperparameters for the RMSProp Optimizer	0.9
momentum	Momentum, hyperparameters of the RMSProp optimizer	0
epsilon	Optimizer stability parameters, small constants	1.00 × 10^−8^

**Table 6:** Difference in distribution between post-fill and measured data.

Indexes	Upper Guide X-Axis Swing	Upper Guide Y-Axis Swing	Head	Active Power
KL dispersion	0.0058	0.0019	0.0022	0.0029
JS dispersion	0.0008	0.0005	0.0006	0.0012
Hellinger distance	0.0126	0.0187	0.0193	0.0703

**Table 7:** Evaluation of the effectiveness of different methods for filling errors in hydropower unit monitoring data.

Parameter	Missing Rate	WSGAIN-GP	SGAIN	GAIN	KNN
upper guide X-axis swing	10%	0.2926	0.3188	0.3409	0.3013
	30%	0.3027	0.4078	0.3576	0.3158
	50%	0.6936	0.8811	0.8090	0.7173
	70%	1.0999	1.2916	1.2445	1.1158
upper guide Y-axis swing	10%	0.2179	0.3780	0.2515	0.2199
	30%	0.2301	0.4044	0.2581	0.2472
	50%	0.6484	0.8135	0.7013	0.6519
	70%	1.0369	1.2503	1.1469	1.0594
active power	10%	0.1779	0.2836	0.2213	0.2038
	30%	0.2060	0.4014	0.2435	0.2557
	50%	0.6650	0.9399	0.7405	0.7633
	70%	1.1561	1.4529	1.2277	1.2772
head	10%	0.2232	0.3485	0.2542	0.2394
	30%	0.3088	0.4406	0.3267	0.3403
	50%	0.5727	0.9153	0.7030	0.6430
	70%	1.0594	1.4229	1.1733	1.1496

## Data Availability

The raw/processed data cannot be shared at this time. Due to the nature of this research, the participants of this study did not agree that their data be shared publicly.
